# Cholesterol Hinders the Passive Uptake of Amphiphilic Nanoparticles into Fluid Lipid Membranes

**DOI:** 10.1021/acs.jpclett.1c02077

**Published:** 2021-09-01

**Authors:** Ester Canepa, Davide Bochicchio, Matteo Gasbarri, Davide Odino, Claudio Canale, Riccardo Ferrando, Fabio Canepa, Francesco Stellacci, Giulia Rossi, Silvia Dante, Annalisa Relini

**Affiliations:** † Department of Chemistry and Industrial Chemistry, University of Genoa, via Dodecaneso 31, 16146 Genoa, Italy; ‡ Materials Characterization Facility, Istituto Italiano di Tecnologia, via Morego 30, 16163 Genoa, Italy; § Department of Physics, University of Genoa, via Dodecaneso 33, 16146 Genoa, Italy; ∥ Institute of Materials, École Polytechnique Fédérale de Lausanne, Route Cantonale, 1015 Lausanne, Switzerland

## Abstract

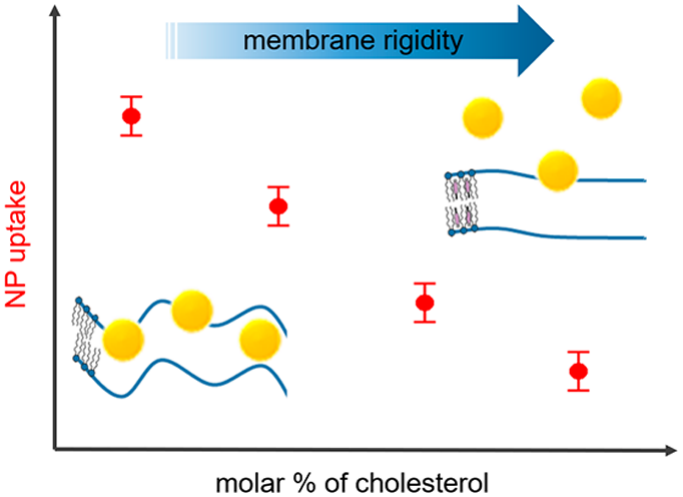

Plasma membranes represent pharmacokinetic barriers for the passive transport of site-specific drugs within cells. When engineered nanoparticles (NPs) are considered as transmembrane drug carriers, the plasma membrane composition can affect passive NP internalization in many ways. Among these, cholesterol-regulated membrane fluidity is probably one of the most biologically relevant. Herein, we consider small (2–5 nm in core diameter) amphiphilic gold NPs capable of spontaneously and nondisruptively entering the lipid bilayer of plasma membranes. We study their incorporation into model 1,2-dioleoyl-*sn*-glycero-3-phosphocholine membranes with increasing cholesterol content. We combine dissipative quartz crystal microbalance experiments, atomic force microscopy, and molecular dynamics simulations to show that membrane cholesterol, at biologically relevant concentrations, hinders the molecular mechanism for passive NP penetration within fluid bilayers, resulting in a dramatic reduction in the amount of NP incorporated.

Cellular uptake is a key process for many delivery strategies assisted by ligand-protected nanoparticles (NPs).^1,2^ However, to reach the intracellular target effectively, NPs must first overcome the plasma membrane barrier. Similar to many drug molecules,^1,3,4^ small NPs with a size comparable to the thickness of the membrane bilayer can passively translocate to the cytosol of living cells.^5−7^ Although rarer than the endocytic pathway,^8^ passive NP internalization has important advantages. The entrapment of NPs in acidic endo/lysosomal vesicles may hinder delivery^9^ and induce in situ degradation of NPs into cytotoxic byproducts.^10^ Carried species must have suitable physicochemical properties to evade the cellular endocytic machinery and gain direct entry into the cell interior.^11^ Balanced hydrophobic and electrostatic effects at the bilayer interface have been revealed as a distinguishing feature for promoting and guiding the passive incorporation of amphiphilic drugs^4,12−16^ or amphiphilic-based drug carriers into cells. This is the case, for instance, for certain cell-penetrating peptides,^17^ polymers,^18,19^ and ligand-protected NPs.^20−22^ When provided with sufficient conformational flexibility,^23^ amphiphilic structures tend to adopt optimized configurations that maximize favorable interactions with the hydrophobic/hydrophilic regions of the lipid bilayer, thus achieving passive diffusion within the membrane.^24^

The efficiency and mechanism of cellular internalization also depend on the properties of the plasma membrane. Among them, the degree of membrane fluidity is known to play a key regulatory function for many cellular processes,^25^ including interaction with exogenous NPs. To date, the impact of membrane fluidity in shaping passive NP uptake has primarily been addressed using biomimetic membrane models composed of phosphocholines (PCs), the main plasma membrane components. At room temperature, amphiphilic ligand-protected NPs with a core size of ∼3 nm were reported to spontaneously penetrate exclusively within fluid-state PC membranes.^26,27^ Consistently, membrane fluidization induced by large temperature increases was reported to dramatically boost the incorporation of NP into high-melting PC bilayers.^26,28^ These results suggest that modulation in membrane fluidity profoundly affects the ability of NPs to passively enter lipid bilayers, yet when considering the structure of mammalian plasma membranes, other intrinsic factors, predominant over temperature changes, determine the degree of membrane fluidity and the process of NP internalization. This is the case for membrane cholesterol content. Cholesterol is arguably the most biologically relevant membrane constituent capable of modulating the functional fluidity of mammalian plasma membranes over a wide temperature range.^29,30^ The membrane cholesterol content ranges from ∼20 to 50 mol % of the total membrane lipids^31^ and differs by cell type and species; high contents, for instance, have been reported for myelin sheaths, astrocytic and neuronal plasma membranes,^32^ and some cancer cells.^33^ While the impact of membrane cholesterol on passive drug incorporation has been the subject of extensive study,^34−39^ the effects of varying cholesterol concentrations on passive binding of ligand-protected NPs to cell membranes have yet to be properly addressed.

This work focuses on monodisperse amphiphilic AuNPs with a mean core size of 2.4 nm (Figure S1) and coated by a mixture of the hydrophilic, negatively charged 11-mercapto-1-undecanesulfonate (MUS) and the hydrophobic 1-octanethiol (OT) (Figure S2). These NPs have attracted biomedical interest due to the biocompatibility of the gold core, the high colloidal stability in aqueous media, and the possibility of being loaded with hydrophobic molecules and used for imaging and therapeutic applications.^40−42^ Most importantly, they have been consistently shown to passively and nondisruptively penetrate the plasma membrane of various mammalian cells *in vivo*(43) and *in vitro*(41,44−46) and fluid phases of PC-based lipid bilayers.^7,26,47,48^ Although extensive efforts have been devoted to elucidating the molecular internalization mechanism of these NPs into PC bilayers,^20,24,49,50^ the impact of cholesterol-induced reduction in membrane fluidity on NP uptake is herein evaluated.

Our investigation relies on a combined experimental and computational approach designed to systematically and quantitatively characterize the ability of cholesterol-containing model cell membranes to incorporate amphiphilic NPs passively. Diunsaturated 1,2-dioleoyl-*sn*-glycero-3-phosphocholine (DOPC) is chosen due to its abundance in mammalian cell membranes and its low fluid–gel phase transition (−17 °C), which makes it suitable for fluid phase studies. Increasing amounts of cholesterol in a typical biological range (0–45 mol %) are incorporated into DOPC membranes (Figure S3) to mimic the varying fluidity of mammalian plasma membranes. Our force spectroscopy measurements based on atomic force microscopy (AFM) show that the mechanical rigidity of DOPC increases with cholesterol content, while quartz crystal microbalance with dissipation monitoring (QCM-D) shows that NP uptake is reduced with cholesterol content. Molecular dynamics simulations provide an interpretation of the experimental results, showing how membrane rigidity causes an increase in the free energy barriers that regulate the incorporation of NPs into the bilayer core.

DOPC bilayers were recently reported to undergo a progressive cholesterol-induced reduction in local fluctuation dynamics, with a concomitant increase in bilayer bending stiffness^51,52^ and packing density.^51^ Here, we prepared supported lipid bilayers (SLBs) via vesicle fusion and acquired AFM tapping-mode images to characterize the topography of the SLB surface, which was found to be uniformly flat on a scale of tens of micrometers regardless of cholesterol concentration (Figure S4). Then, we performed AFM-based force spectroscopy measurements to check the variation in membrane fluidity before interaction with NPs. Our AFM nanomechanical investigation indicated a clear increase in the bilayer Young’s modulus (Figure 1A) and breakthrough force (Figure S5) with an increase in cholesterol content. Such an effect was further investigated by steady-state fluorescence anisotropy measurements on vesicles labeled with the hydrophobic fluorophore 1,6-diphenyl-1,3,5-hexatriene (DPH).^53,54^ As shown by Figure 1B, DPH anisotropy increased linearly upon addition of cholesterol, thus confirming the cholesterol-induced packing effect on bilayer acyl chains.

**Figure 1:**
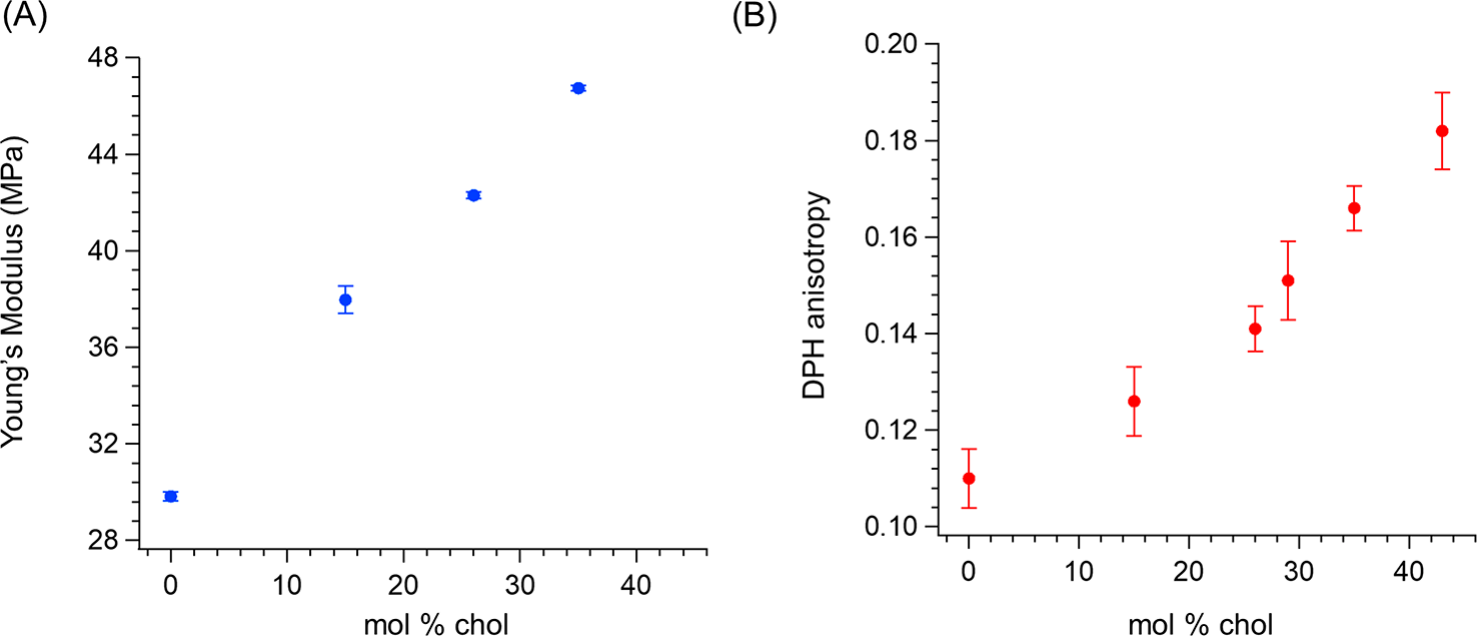
Progressive stiffening of the fluid DOPC bilayer induced by increasing membrane cholesterol concentrations. (A) AFM nanomechanical results on the SLB Young’s modulus. (B) Fluorescence anisotropy emission (eq 1, Supporting Information) of the DPH fluorophore embedded in the vesicle bilayer.

The stiffening of DOPC bilayers due to cholesterol is reproduced by both atomistic^51,55^ and coarse-grained (CG)^56^ computational models. Qualitatively, all models predict a progressive increase in membrane rigidity with an increase in cholesterol content. We investigated the impact of this increased rigidity on NP incorporation by using a CG model based on the MARTINI force field,^57^ as reported in several previous studies.^24,27,50^ The CG structures of the ligands and the complete 70:30 MUS:OT NP (gold core diameter of 2 nm) used in this work are shown in Figure 2A. Because of the sampling advantages given by the fast dynamics of the CG model, it is possible to observe the spontaneous penetration of a NP into the lipid bilayer core.^24^ NP incorporation has been deeply investigated in previous studies,^24,50^ and it is a multistep process (Figure S6). In the first step, the NP simply adsorbs to the membrane. Then, it proceeds to a metastable state in which there are contacts between the hydrophobic groups of the NP ligands and the lipid tails (hydrophobic contact state). Finally, the proper NP incorporation takes place in a third step. One by one, the MUS ligands translocate the hydrophobic core of the bilayer and anchor their termini to the headgroups of the distal leaflet. The first ligand anchoring (see Figure 2B) can be considered as the key step after which the NP is stably bound to the membrane. Therefore, we designed unbiased CG MD simulations to investigate how cholesterol-induced membrane stiffening influences this process.

**Figure 2:**
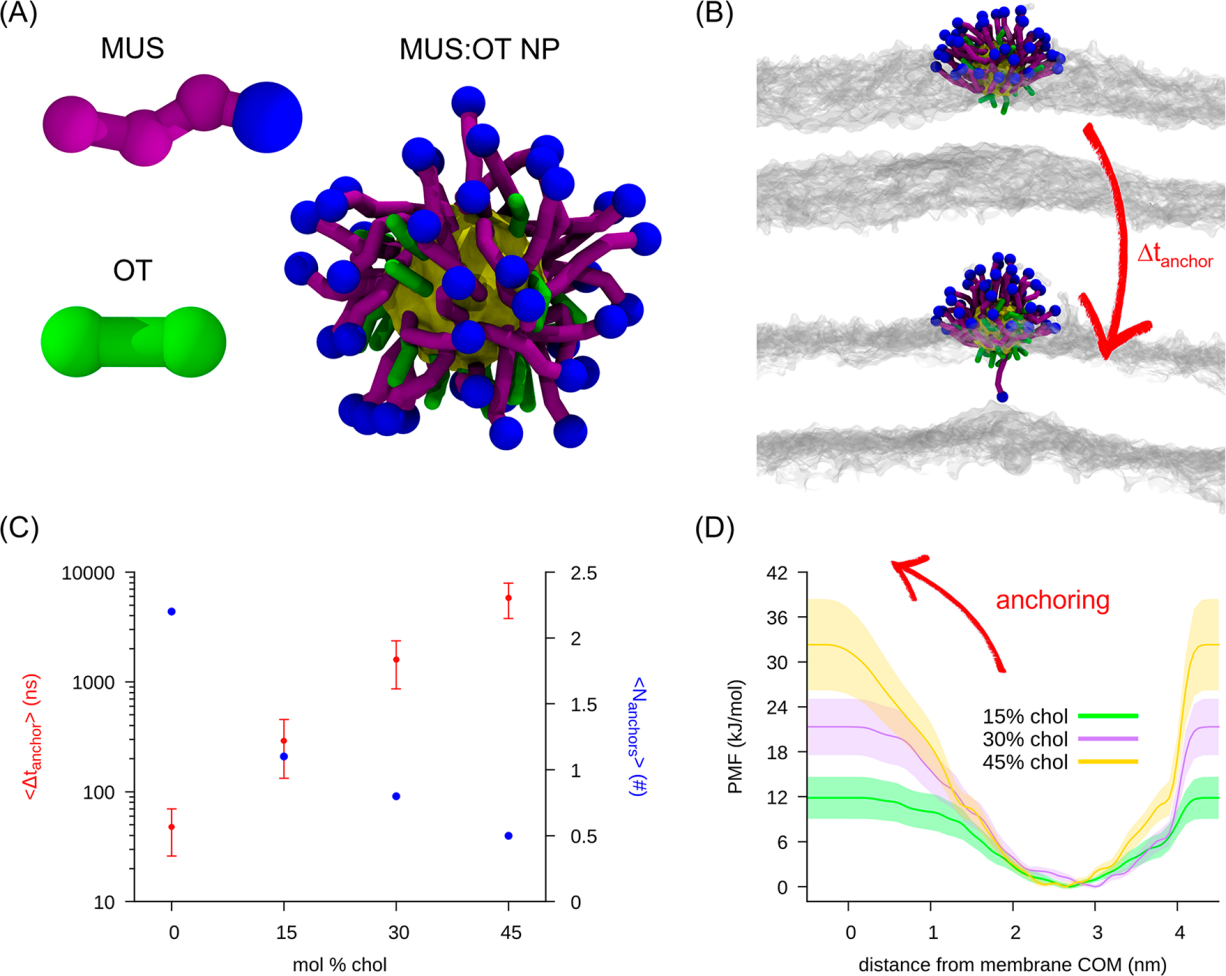
Computational investigation of the effect of cholesterol on the uptake of a MUS:OT NP in DOPC membranes. (A) Coarse-grained structure of the hydrophilic (MUS) and hydrophobic (OT) ligand, together with the typical structure of a 70:30 MUS:OT NP (gold core diameter of 2 nm) in water (water not shown). Blue beads are the negatively charged termini of the MUS ligands. (B) Simulation snapshots illustrating the ligand anchoring process. The NP goes from the hydrophobic contact state (top) to the anchored state (bottom) in which one MUS charged terminal is in contact with the DOPC heads (transparent gray) of the distal leaflet. (C) Average anchoring time (Δ*t*_anchor_) and average number of anchored ligands after 1 μs (*N*_anchors_) as a function of the mole percent of cholesterol, obtained from unbiased MD simulations. (D) Anchoring free energy barriers at different mole percents of cholesterol, obtained from WT-MetaD simulations.

We prepared and equilibrated DOPC lipid bilayers containing different molar percentages of cholesterol [0%, 15%, 30%, and 45% (see the Supporting Information for computational details)], solvated in water and 150 mM NaCl. Then, we inserted a single NP (gold core radius of 2 nm) in water and let it spontaneously diffuse, adsorb, and eventually enter the intermediate hydrophobic contact stage (Figure 2B, top). We ran 12 independent simulations for cholesterol content measuring the time interval spent by the NP in the hydrophobic state until the anchoring of the first ligand [Δ*t*_anchor_ (Figure 2B, bottom)]. The average Δ*t*_anchor_ for each system is reported in Figure 2C. The first anchoring event is progressively slowed by increasing the cholesterol content. The slowdown is not restricted to the anchoring of the first ligand, as one can notice by the average number of anchored ligands after 1 μs [*N*_anchors_ (reported in Figure 2C)]. While *N*_anchors_ is >2 for a pure DOPC membrane, it decreases to 0.5 in the case of 45% cholesterol, and its decrease is inversely proportional to the cholesterol content. Interestingly, the average Δ*t*_anchor_ increases exponentially with cholesterol content, consistently with a single anchoring free energy barrier increasing with cholesterol content. This point is confirmed by the cumulative distributions of the anchoring times (Figure S7). The cumulative Poissonian distribution fits well the Δ*t*_anchor_ distributions obtained from our unbiased simulations: the probability of observing at least one anchoring event [*P*_(*n*=1)_] within a time *t* is well approximated by ,^58^ where τ is the characteristic transition time (Figure S7). Here it is worth recalling that the time scale of anchoring events in CG simulations is largely underestimated: it should not be directly compared to the experimental time scale, which is on the order of 10–10^4^ s. More quantitative reasoning about the rescaling of the CG time to the laboratory time can be found in the Supporting Information.

We performed well-tempered metadynamics (WT-MetaD) simulations to quantify the free energy barriers associated with the rare anchoring events.^59^ We biased the distance between the charged terminal of a MUS ligand and the membrane center, as discussed in detail in previous works.^24,50^ The WT-MetaD setup is described in the Supporting Information. We performed eight independent WT-MetaD simulations for each system containing cholesterol. The results are reported in Figure 2D. The anchoring barrier increases from ∼12 kJ/mol (at 15% cholesterol) to ∼21 kJ/mol (30% cholesterol) to ∼32 kJ/mol (45% cholesterol). The linear increase in the barrier with cholesterol content is consistent with the exponential growth of Δ*t*_anchor_ observed in unbiased MD simulations.

Our computational results consistently indicate that the stable incorporation of NPs is slowed in the presence of cholesterol and that this slowdown accelerates with an increase in cholesterol content. In the following section, we experimentally validate this computational prediction employing QCM-D measurements.

Lipid vesicles were deposited in phosphate-buffered saline (PBS) on gold-coated QCM sensors where they adsorb without merging. In this way, smooth supported vesicle layers (SVLs) with viscoelastic properties were formed^60^ and then incubated with AuNPs. Throughout the experiment (outlined in Figure S8), changes in frequency (Δ*f*) and dissipation (Δ*D*) were recorded in real time to monitor vesicle integrity and quantify spontaneous NP incorporation within the vesicle bilayer. Figure 3A shows the frequency shifts for SVL formation at varying cholesterol concentrations (see Figure S9 for the concomitant increase in Δ*D*). In general, the adhesion of the vesicle to the sensor occurred within a few hours, as indicated by a plateau in the frequency curve (Δ*f*_SVL_). Notably, the SVL formed more rapidly and caused a larger Δ*f*_SVL_ as the cholesterol concentration increased. Due to the SVL viscoelasticity, QCM-D data were quantitatively interpreted considering potential variations in the layer viscoelastic properties, i.e., density, thickness, viscosity, and elasticity, caused by differences in vesicle size and lipid composition.^61^ Here, vesicle size after extrusion did not vary significantly (Figure S3). A viscoelastic model based on the Voigt theory^62^ was thus applied to QCM-D data to determine the contribution of bilayer composition.^61^ Such data processing, whose results appear in Figure 3B and Figure S10, revealed that the SVL density, viscosity, and elastic modulus increased with cholesterol content, whereas the thickness remained unchanged. In the case of pure DOPC vesicles, the layer density reported in Figure 3B (0.762 ± 0.019 g/cm^3^) is in excellent agreement with literature data.^61^

**Figure 3:**
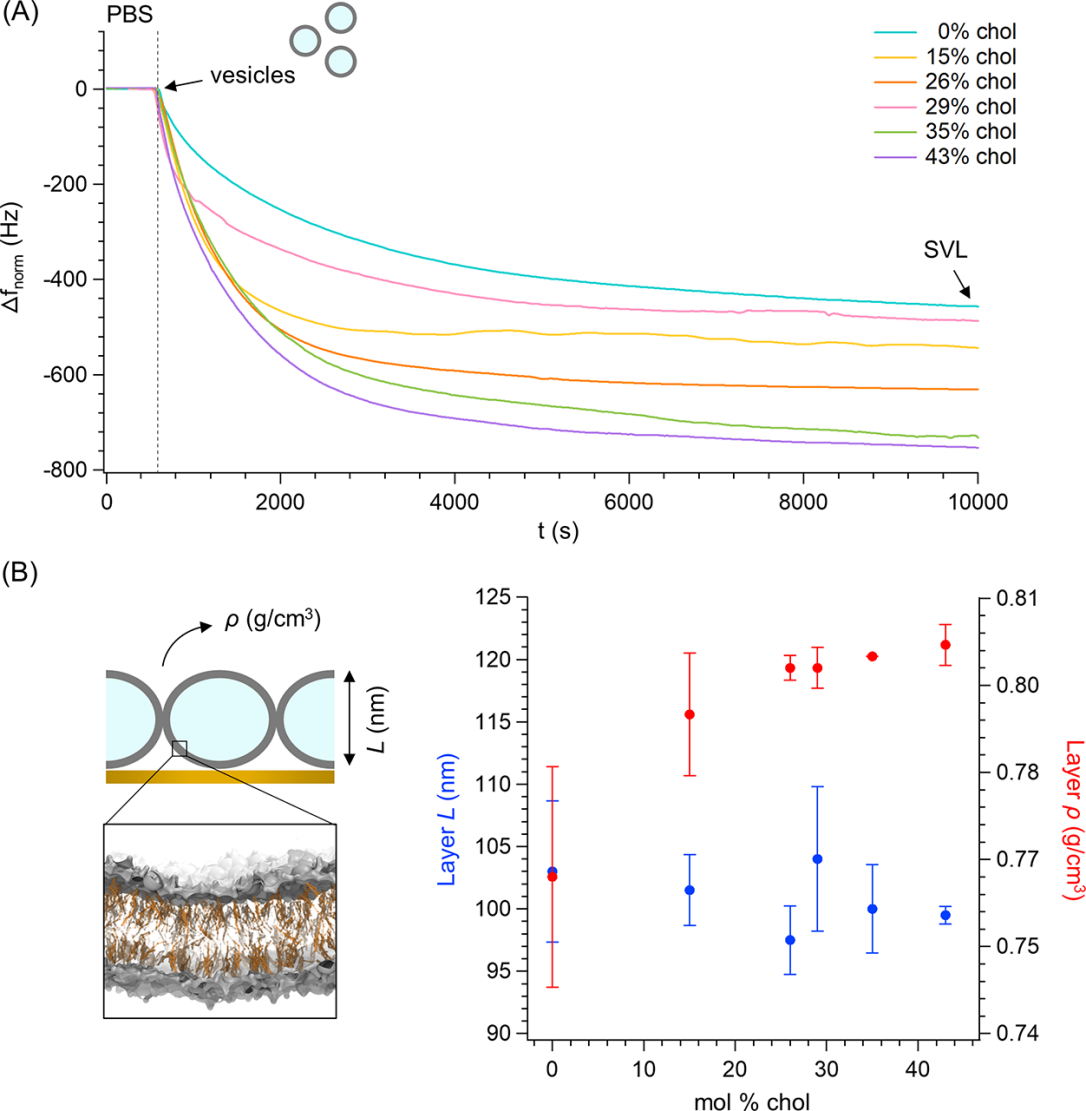
SVL formation in PBS at 22 °C monitored by QCM. (A) Asymptotic frequency shifts (fifth overtone) recorded after the injection in the sensor chamber of lipid vesicles with varying cholesterol molar percentages; Δ*f* was normalized by *n*^1/2^, which is typical when dealing with changes in layer viscosity (see Figure S10). An average over 20 points was applied to real-time frequency data. (B) SVL cartoon (not to scale) and simulation snapshot of the DOPC/chol membrane with 30% chol (left). Lipid heads colored gray (surface representation), lipid tails shown as light gray sticks, and cholesterol shown as tan sticks (lipid tails are made transparent to make tan cholesterol visible). Layer density (ρ) and thickness (*L*) (right), derived from Voigt modeling (see the Supporting Information), as a function of cholesterol content. The layer areal mass is given by the product *Lρ*.^61^

Because both the vesicle diameter after extrusion (Figure S3) and the SVL thickness (Figure 3B) can be considered constant from one bilayer composition to another, it is possible to assume that the number of vesicles per unit area of the sensor was the same for all SVLs. Furthermore, the steady frequency recorded after SVL formation (Figure 3A) indicates no significant loss of vesicles from the sensor surface. These assumptions allow the same overall aqueous content for all SVLs to be considered. Thereby, the increasing Δ*f*_SVL_ values of Figure 3A are explained by the increase in the density, viscosity, and elastic modulus of the deposited layer. This result is consistent with the nanomechanical characterization shown in Figure 1 and structural measurements indicating a cholesterol-induced increase in the level of lipid packing of DOPC/chol bilayers.^51,63^

After being gently rinsed, SVLs were incubated with NPs for at least 20 h at 22 °C. A representative experiment is reported in Figure 4A (see Figures S11 and S12 for additional traces). At all cholesterol percentages, Δ*f* slowly decreased after the addition of NPs until a plateau was reached and no further NP incorporation was possible (Figure S12). Furthermore, the time recorded for Δ*f* flattening (Figure 4A) is consistent with our previous AFM investigation disclosing diffuse incorporation of similar amphiphilic NPs into DOPC-based fluid membranes after incubation of NPs and membranes for 4 h.^27^

**Figure 4:**
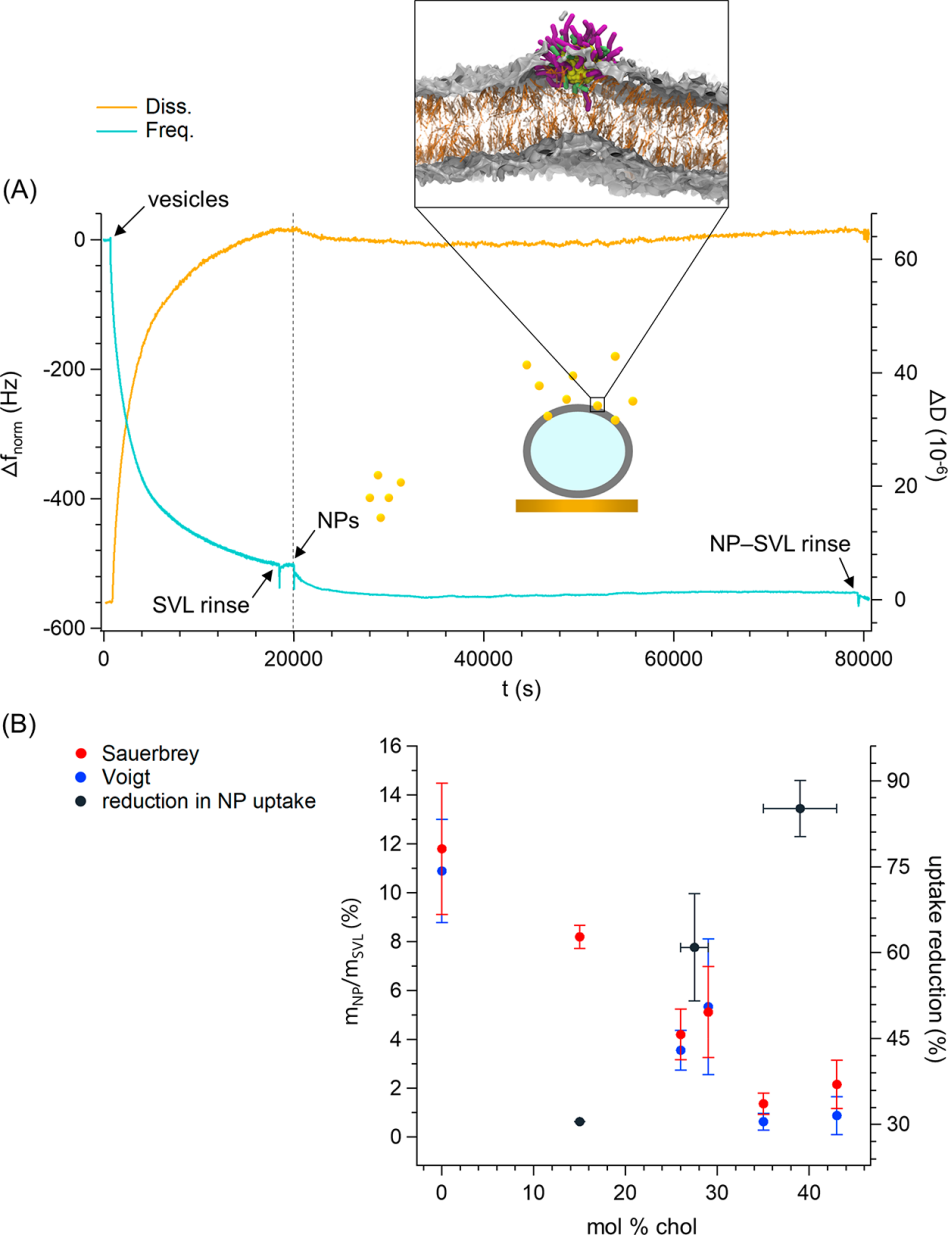
NP-SVL incubation in PBS (22 °C) under QCM-D monitoring. (A) Frequency (Δ*f*) and dissipation (Δ*D*) traces of a representative experiment (0 mol % chol) before and after the addition of NPs. The continuous increase in energy dissipation after vesicle injection is consistent with the formation of a viscoelastic vesicle layer. The SVL was rinsed before the injection of NPs and at the end of the recording; in general, no destabilization was observed in either event. Simulation snapshot: NP color code as in Figure 2 (negatively charged termini of the MUS ligands not highlighted in blue) and bilayer color code as in Figure 3. (B) Percent mass changes of the SVL after maximum NP uptake. Data were calculated using both a modified Sauerbrey equation (eq 2, Supporting Information) and Voigt modeling (see the Supporting Information for full details). The reduction (percent) in NP uptake was normalized with respect to the maximum uptake efficiency in the absence of cholesterol.

In general, NPs did not show significant disruptive behavior during passive bilayer penetration. Only in a few experiments a slight and slow increase in Δ*f* was recorded after incubation for several hours (Figures S11 and S12). However, this event always occurred after Δ*f* stabilized at a minimum value for several minutes. Figure 4B reports the percentage change in the SVL mass after NP incorporation [(*m*_NP_/*m*_SVL_) × 100, where *m*_NP_ is the net mass change due to maximum NP uptake and *m*_SVL_ is the SVL mass before the addition of NPs]. *m*_NP_ and *m*_SVL_ values were calculated separately using two models, i.e., the same Voigt model^61,62^ used to extract the SVL viscoelastic properties before the addition of NPs and a rigid model (Sauerbrey) in which the intrinsically underestimated mass uptake was compensated by normalizing the frequency overtones by *n*^1/2^ (see the Supporting Information for details of the analysis). After NP uptake, all frequency and dissipation shifts were analyzed at the plateau, before any vesicle destabilization. Therefore, NP uptake quantification always assumed that the SVL was unaffected in terms of vesicle number and water content. As shown by Figure 4B, both models provided very similar NP uptake efficiencies. As the membrane cholesterol content increases, the amount of NPs penetrating the unsaturated bilayer decreases linearly up to approximately 35% cholesterol and then stabilizes. Furthermore, the percent mass changes shown in Figure 4B were converted into lipid/NP ratios as detailed in Table S3. In the absence of cholesterol, the result (∼73 lipid/NP) is perfectly consistent with the quantification reported in our previously mentioned investigation of another DOPC-based fluid membrane (∼79 lipid/NP).^27^ By relying on an established lipid system to study NP–membrane interactions,^64,65^ we found that these QCM-D results show an exceptional agreement with our *in silico* investigation and quantify the ability of membrane cholesterol to reduce the passive uptake of amphiphilic NPs endowed with surface conformational flexibility.

In summary, in this paper we have shown that the addition of membrane cholesterol to fluid vesicles decreases the spontaneous uptake of amphiphilic NPs with a diameter of 2.4 nm. The embedding of small NPs into the membrane core takes two players: flexible NP ligands, which can adapt to the water environment and to the hydrophobic membrane core, and membrane fluidity. From a molecular perspective, the formation of a stable NP–membrane complex is triggered by local and rare alterations of the membrane compactness, such as in-plane, out-of-plane, and orientational^66^ lipid fluctuations opening the way to ligand translocation. Cholesterol hinders these lipid dynamics,^56^ thus reducing NP uptake. These results suggest that the passive incorporation of NPs could be tuned, *in vitro*, by modulating any of the controlling factors of membrane fluidity, such as temperature, lipid composition, leaflet composition asymmetry, and membrane protein concentration and type. From an opposite perspective, the same factors should be considered when interpreting cell-specific NP uptake *in vivo*.

## Supporting Information Available

Materials, synthesis of amphiphilic gold nanoparticles (AuNPs), characterization of AuNPs (TEM, DLS, ζ potential, and NMR analyses), preparation of fluid lipid vesicles and supported lipid bilayers (SLBs), DLS characterization of fluid lipid vesicles, details of the setup and analysis of atomic force microscopy (AFM), additional AFM-based measurements (force spectroscopy and tapping-mode imaging), details of the setup and analysis of fluorescence anisotropy, computational methods (coarse-grained models, MD simulation parameters, unbiased simulation settings, well-tempered metadynamics parameters, and rescaling of the CG simulation time), details of the setup and analysis of dissipative quartz crystal microbalance (QCM-D), and additional QCM-D results (characterization of dissipation traces and viscoelastic properties) (PDF)
