# Supplementary material for: Cholesterol Hinders the Passive Uptake of Amphiphilic Nanoparticles into Fluid Lipid Membranes
Source: J Phys Chem Lett. 2021 Sep 1;12(35):8583–90. doi: 10.1021/acs.jpclett.1c02077 (PMC8436204; doi:10.1021/acs.jpclett.1c02077)
Supplement: Supplementary file 1 — jz1c02077_si_001.pdf [file jz1c02077_si_001.pdf]

Supporting Information

Cholesterol Hinders Passive Uptake of Amphiphilic Nanoparticles into Fluid Lipid Membranes

Ester Canepa,[†,‡,¶] Davide Bochicchio,[¶] Matteo Gasbarri,[§] Davide Odino,[¶] Claudio Canale,[¶] Riccardo Ferrando,[¶] Fabio Canepa,[†] Francesco Stellacci,[§] Giulia Rossi,*[¶] Silvia Dante,*[‡] and Annalisa Relini[¶]

[†] Department of Chemistry and Industrial Chemistry, University of Genoa, via Dodecaneso 31, 16146 Genoa, Italy

[‡] Materials Characterization Facility, Italian Institute of Technology, via Morego 30, 16163 Genoa, Italy

[¶] Department of Physics, University of Genoa, via Dodecaneso 33, 16146 Genoa, Italy

[§] Institute of Materials, École Polytechnique Fédérale de Lausanne, route cantonale, 1015 Lausanne, Switzerland

Corresponding Authors

silvia.dante@iit.it

rossig@fisica.unige.it

Materials

Nanoparticles. Hydrogen tetrachloroaurate (III) trihydrate ($HAuCl_4 \cdot 3H_2O$, 0.2 % max. alkalis and other metals), oleylamine (C18-content 80-90 %), and *n*-octane (97 %) were purchased from Acros Organics (Fisher Scientific). *Tert*-butylamine borane complex (tBAB, powder, 97 %), 1-octanethiol (OT, ≥ 98.5 %), ethanol absolute (EtOH, > 99.8 %), dichloromethane (DCM, ≥ 99.8 %), acetone (≥ 99.9 %), tetrahydrofuran (THF, ≥ 99.9 %), and deuterated solvents were purchased from Sigma Aldrich (Merck). All chemicals were used without further purification unless otherwise stated. The anionic 11-mercapto-1-undecanesulfonate (MUS) ligand was prepared in house according to a previously reported protocol[1].

Lipid vesicles. 1,2-dioleoyl-*sn*-glycero-3-phosphocholine (DOPC, 18:1(Δ9-Cis) PC, > 99 %) was purchased as lyophilized powder from Avanti Polar Lipids. Cholesterol (chol, ≥ 99 %, lyophilized powder), 1,6-diphenyl-1,3,5-hexatriene (DPH, 98 %), chloroform ($CHCl_3$, ≥ 99.5 %), methanol (CH_3OH, ≥ 99.9 %), phosphate buffered saline (PBS, 1x, without calcium chloride and magnesium chloride, pH 7.4) were purchased from Sigma Aldrich (Merck). All chemicals were used without further purification.

Before use, water was always purified with a Milli-Q ultrapure water system (18.2 MΩ·cm resistivity at 25 °C; Merck Millipore).

Synthesis of monodisperse amphiphilic AuNPs

Amphiphilic AuNPs with low core size dispersion (i.e. monodisperse AuNPs) were prepared applying few minor modifications to the procedure published by Yang et al.[2] This procedure is divided into two steps: **1)** synthesis of monodisperse oleylamine (OAm)-coated AuNPs and **2)** complete thiol-for-OAm ligand-exchange.

1) OAm-AuNP synthesis. All glassware was washed in *aqua regia* before use. A magnetic hotplate stirrer equipped with temperature control was used to stir and heat the reaction mixture. Temperature gradients were avoided by immersing the reaction flask in a thermostated water bath. In a 100 ml 3-neck round-bottomed glass flask, $HAuCl_4 \cdot 3H_2O$ (0.5 mmol) was dissolved in 16 mL OAm and 20 mL *n*-octane. The flask was sealed with rubber septums, abundantly flushed with Ar to exchange all air, and placed in a sonicator bath for ca. 1 min before stirring; a static Ar atmosphere was then maintained throughout the synthesis. The reaction temperature was set to 45 °C. After temperature equilibration, a tBAB reducing solution (0.5 mmol in 4 mL of OAm) was quickly added to the gold-OAm mixture. After one hour, ca. 40 mL of EtOH was added to quench the reaction and

facilitate NP precipitation. Final OAm-AuNPs were collected by centrifugation at 5000 g for 10 min. Three more washing cycles in EtOH were performed (a small volume of DCM was used to redisperse the product). Final OAm-AuNPs were dried under high vacuum to obtain shiny dark powders.

2) Thiol-for-OAm ligand exchange. The ligand exchange was performed at room temperature. In separate glass vials, ca. 30 mg of hydrophobic OAm-AuNPs and a large excess (0.1 mmol) of the thiol mixture were dispersed in DCM (5 mL and 20 mL, respectively). To obtain a final 2:1 MUS:OT molar ratio in the ligand-shell, the stochiometric thiol ratio in solution was set to 3:2. The thiol mixture was sonicated for several minutes before adding, under vigorous magnetic stirring, the NP dispersion to start the ligand-exchange. During the first 2 h, the exchange mixture was mildly sonicated for ca. 30 s every half hour; the stirring was then left overnight. The same sonication procedure was repeated 2 h before NP washing. After ~12-15 h, the exchange mixture was diluted in acetone (ca. 15 mL) and centrifugated (5000 g, 4 min). To remove all hydrophobic unbound ligands (OAm and excess OT), 6 washing cycles in organic solvent were performed before vacuum drying. Dried AuNPs were then redispersed in water (ca. 15-20 mL) and centrifuged (4000 g, 6 min) in a hydrated AMICON® ultra centrifugal tube (regenerated cellulose membrane, 10 kDa cutoff molecular weight) to filter off the excess of water-soluble MUS (this procedure was repeated 15x). The purified NP aqueous dispersion was freeze-dried to obtain a manageable NP powder. Before characterization, 2:1 MUS:OT AuNPs were suspended in water and diluted in the experimental buffer (PBS) when specified. In general, they showed remarkable long-term colloidal stability in both aqueous media and their dispersion did not require further manipulation before use.

Preparation of lipid vesicles with tunable bilayer stiffness

Increasing cholesterol amounts were added to DOPC to prepare fluid lipid vesicles with increased bilayer stiffness. Lyophilized DOPC was weighed in a glass vial, dissolved in $CHCl_3$:CH_3OH (2:1, v/v), and divided into aliquots. Stochiometric amounts of cholesterol – previously dissolved in the same solvent mixture – were added to DOPC aliquots to set the chol mol % to 17, 30, 33, 40, and 50 %. After gently mixing to homogenize each sample, the solvent was evaporated under a stream of N_2. After ca. 24 h under vacuum, the lipid films were then hydrated in PBS at a 2 mg/mL lipid concentration. To promote the formation of multilamellar vesicles, hydrated samples were sonicated in an ultrasonic bath for ca. 15 min at room temperature. Subsequently, always at room temperature, each lipid suspension was extruded 25 times using the Avanti Mini-Extruder (Avanti Polar Lipids) with a 0.1 μm pore diameter polycarbonate membrane (Nuclepore filters, Whatman). For fluorescence anisotropy assays, the vesicle bilayer was labeled by adding small aliquots of DPH to

the organic DOPC/chol mixtures (1:1000 probe-to-lipid molar ratio[3]). A DPH stock solution was previously prepared in $CHCl_3$:CH_3OH (2:1, v/v). Such relatively low DPH molar fraction was chosen to yield adequate signal to noise ratio and avoid any probe-induced perturbation of the bilayer structure. Upon hydration of labeled lipid films, DPH is known to intercalate within the lipid bilayer[4]. All vesicle suspensions were stored at 4 °C and used within a few days; batches containing fluorescent probes were also protected from light till further use.

Estimated cholesterol content into the extruded DOPC bilayer. It is known that the vesicle phospholipid:chol composition is not equal to the lipid ratio before film hydration. In particular, the actual cholesterol content in the vesicle bilayer is always lower than the nominal content added in solution, and in general, the gap between the two values increases with increasing cholesterol. A robust and accurate determination of cholesterol content in DOPC/chol vesicles extruded as in this work has already been reported by Goñi et al.[5] Based on the incorporation efficiency reported in that study, the cholesterol content in the vesicles used in this work was estimated as shown in Table S1.

Table S1. Average estimated cholesterol content in DOPC/chol vesicles extruded using Nuclepore filters (0.1 μm pore diameter) at room temperature and pH 7.4.

Nominal mol % chol	*Estimated mol % chol*
17	**15**
30	**26**
33	**29**
40	**35**
50	**43**

AuNP characterization

Electron microscopy analysis. Bright-field transmission electron microscopy (BF-TEM) characterization was perfomed on MUS:OT AuNPs using a FEI Tecnai Osiris operated at 200 kV. After sonication in an ultrasonic bath, few drops of a diluted NP water dispersion were deposited onto an ultrathin carbon-coated Cu grid. A TEM image showing monodisperse spherical NPs is reported in Figure S1. The core size distribution (Table S2) was calculated by assuming spherical morphology and by automatically counting a few thousand (at least 1000) particles with ImageJ software.

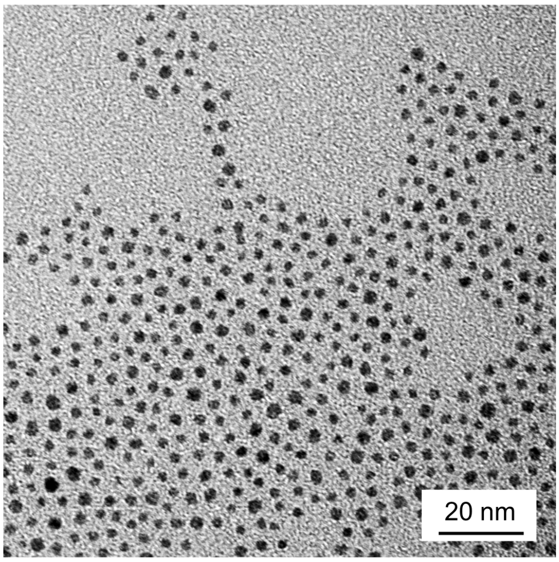

Figure S1. Bright-field TEM image of monodisperse 2:1 MUS:OT AuNPs used in the experiments. Core diameter: 2.4 ± 0.4 nm (average ± one standard deviation).

DLS and ζ-potential analyses. The hydrodynamic size of MUS:OT AuNPs in aqueous dispersion was measured by dynamic light scattering (DLS). In addition, NP ζ-potential was evaluated from electrophoretic mobility measurements to assess the colloidal stability of NP dispersions before interaction with lipid membranes. For both hydrodynamic size and ζ-potential measurements, a Malvern Zetasizer Nano ZS instrument (scattered light collected in backscattering at 173°) was used. Measurements were performed in water and the experimental buffer (PBS); results are reported in Table S2. NP concentrations of 0.3 mg/mL and 0.2 mg/mL were used for size analysis in water and in buffer, respectively, whereas ζ-potential acquisitions were performed at 0.07 mg/mL in water and 0.1 mg/mL in buffer. In all cases, NP dispersions were sonicated in an ultrasonic bath for ca. 30 seconds before analysis.

^{1}H NMR analysis. Proton nuclear magnetic resonance (^{1}H NMR) characterization was performed on MUS:OT AuNPs using a Bruker AVIII400 UltraShield Plus (400 MHz for ^{1}H) spectrometer. ^{1}H NMR analysis was first used to verify the absence of unbound ligands (OAm and excess thiols). When ligands are grafted onto the NP surface, they only generate broadened signals due to interference by the gold core, thus preventing quantitative interpretation of the integrated peaks[1]. For this control, NPs (4-5 mg) were dispersed in D_2O (800 µL), sonicated for ca. 20 min, transferred into a 5 mm NMR tube, and analyzed. The absence in the ^{1}H NMR spectrum (Figure S2A) of sharp peaks generated by free molecules indicates that no unbound ligands were present[1]. Subsequently, the Au core was etched by a large iodine excess to quantify the MUS:OT molar composition in the ligand shell. This procedure induces the decomposition of the gold core by

releasing all thiol ligands as free disulfides[1]. A concentrated iodine solution was prepared by dissolving 15-20 mg of iodine in 800 µL CD_3OD. After ca. 20 min of sonication to favor complete iodine solubilization, 800 µL of the etchant solution was added to NPs (4-5 mg). The NP-iodine mixture was sonicated in an ultrasonic bath for approx. 30 min before transferring the light orange supernatant to a 5 mm NMR tube to acquire the spectrum (Figure S2B). No residual OAm trace was detected, thus confirming that the thiol-for-OAm ligand exchange was complete. After normalization on the number of nuclei and correction due to each contribution, the integral values indicated in Figure S2B were used to calculate the MUS:OT molar ratio (full details of ligand ratio calculation are described in ref[1]). Final ligand composition is reported in Table S2.

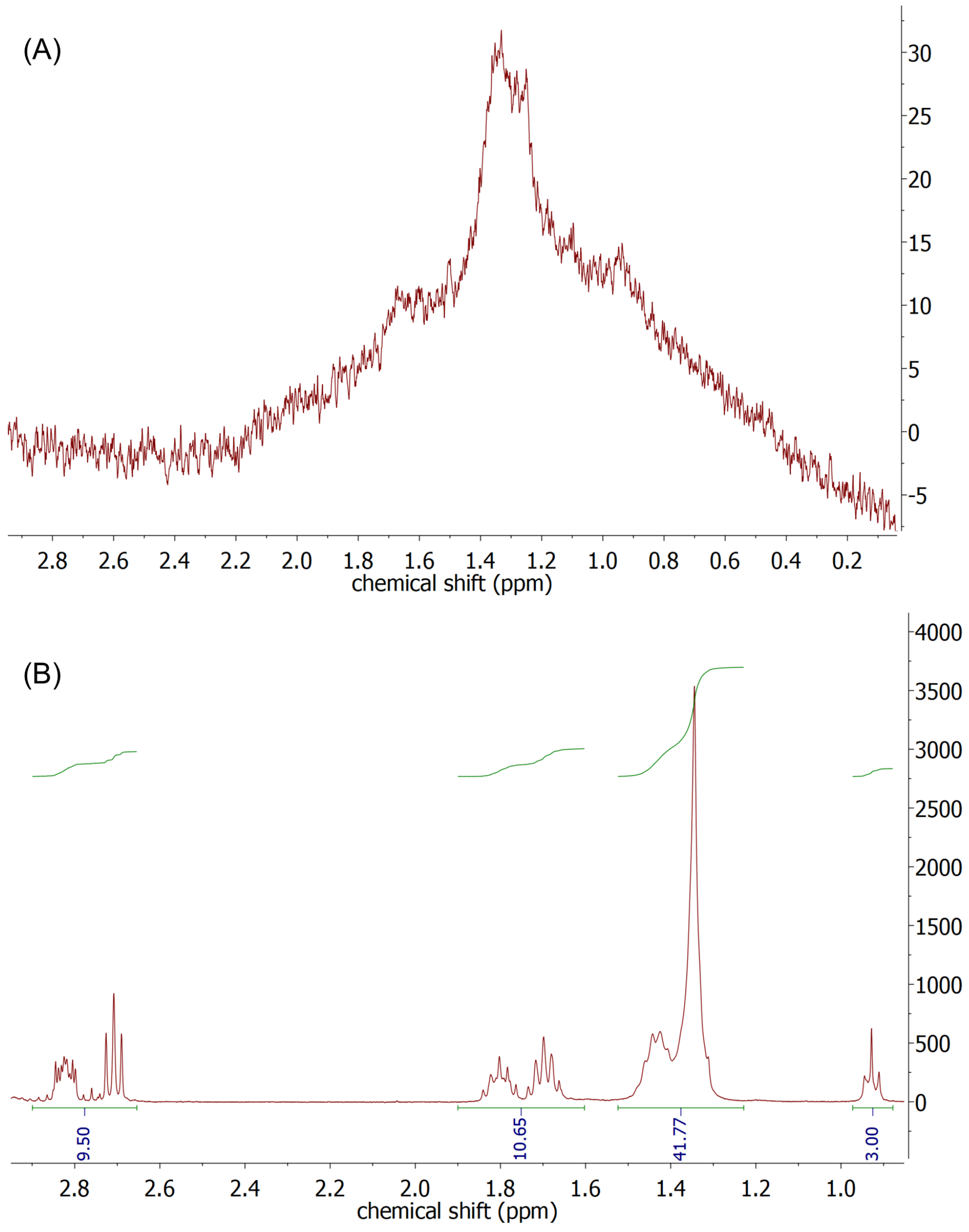

Figure S2. ^1H NMR spectra of 2:1 MUS:OT AuNPs (A) before and (B) after decomposition of the gold core by iodine-etching. ^1H NMR spectra were acquired with 32 scans; post-acquisition

processing was performed with MestReNova (Mestrelab research v. 11.0): automatic phase correction, full auto (polynomial fit) baseline correction, and manual integration.

Table S2. Characterization of monodisperse 2:1 MUS:OT AuNPs used in the experiments.

TEM	*^{1}H NMR*	*DLS hydrodynamic size (nm)* [c]		*ζ-potential (mV)* [c]	
core size (nm) [a]	*% of OT* [b]	*water*	*PBS*	*water*	*PBS*
2.4 ± 0.4	32 ± 5	5.6 ± 1.7	11.2 ± 0.4	−46 ± 5	−38 ± 2

a. mean diameter ± one standard deviation.

b. average (mol %) ± error calculated using Student's statistics (95 % confidence level); three NMR spectra were repeated on separately weighed NP samples.

c. mean value ± error calculated using Student's statistics (95 % confidence level, N=12 – 18); the hydrodynamic size was averaged from number distributions.

NOTE: DLS and ζ-potential values recorded in PBS and reported in Table S2 clearly indicate that the final NP ligand composition – i.e. 2:1 MUS:OT (molar ratio) – provided high colloidal stability in the experimental conditions used for QCM-D measurements.

Lipid vesicle characterization

DLS analysis. As for NPs, DLS measurements were performed at room temperature using a Malvern Zetasizer Nano ZS instrument with a 173° detection angle (Malvern Instruments) to characterize the vesicle hydrodynamic size (Figure S3). Measurements were carried out right after extrusion, without diluting the final vesicle suspensions.

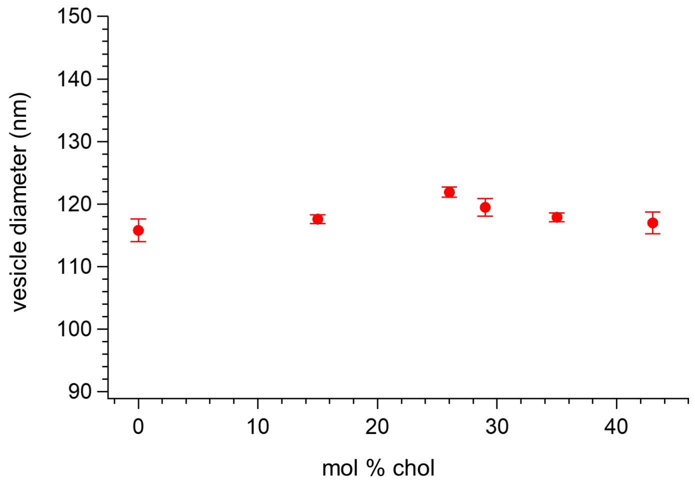

Figure S3. Vesicle diameters (average ± uncertainty) after extrusion, at varying membrane cholesterol contents. Data were obtained from intensity distributions Uncertainties on average values were obtained using Student's statistics, assuming a confidence level of 95 % (N=6).

AFM measurements

AFM measurements were performed on SLBs in the absence of NPs, at room temperature and in liquid environment., Experiments were developed as follows:

- contact-based Quantitative Imaging (QI^{TM} by JPK) was exploited to map the Young's modulus of SLBs with increasing membrane stiffness (Figure 2A, main text). Tapping mode imaging was also performed to obtain topographic images at higher resolution (Figure S4). Measurements were carried out in PBS using a Nanowizard III AFM (JPK Instruments) mounted on an Axio Observer D1 inverted optical microscope (Carl Zeiss). The drive frequency was 12-14 kHz, the scan rate 0.5-1.5 Hz.
- contact-based Force Volume imaging was performed to investigate the cholesterol-induced variation in the SLB breakthrough force (Figure S5). Measurements were carried out in water using a Multimode SPM equipped with "E" scanning head (maximum scan size 15 μm) and driven by a Nanoscope V controller (Digital Instruments-Bruker).

In both cases V-shaped silicon nitride cantilevers (DNP-10, cantilever C, Bruker, nominal spring constant: 0.24 N/m) were used.

Sample preparation. For tapping and QI^{TM} mode measurements, freshly extruded DOPC vesicles with varying cholesterol content were diluted in PBS (40 μL, 0.1 mg/mL) and deposited on silicon wafers of approx. 1.0×1.0 cm^2. The silicon wafers had been pre-treated with UV/Ozone for at least 10 min. After vesicle deposition, samples were stored for 10 min at room temperature and then incubated for 15 min at 60 °C in a close chamber at 100 % relative humidity. On silicon, vesicles tend to merge helped by the increase in temperature and form a supported lipid bilayer (SLB). The SLBs were allowed to cool to room temperature and finally rinsed gently with water. This step was necessary to remove undeposited vesicles from the liquid that may interfere with AFM measurements. For breakthrough force experiments only, lipid vesicles were diluted in water (40 μL, 0.1 mg/mL) and deposited on an approx. 1.0×1.0 cm^2 freshly cleaved mica foil. On mica, a fresh $CaCl_2$ solution (10 μL, 10 mM) was added immediately after vesicle deposition to facilitate vesicle fusion. The other steps of the SLB deposition protocol remained unchanged. In general, AFM measurements started at least two hours after rinsing to allow the system to equilibrate.

Force measurements. QI images (5×5 μm^2 scan size) based on 128×128 force curves were collected after cantilever calibration with the thermal noise method[6]. Very short force curves, with low tip-sample interaction, were acquired for each scan point to obtain small bilayer indentations – setpoint or loading force: 0.6 nN; Z length: 50 nm; speed 10 μm/s; data acquisition rate: 200 kHz;

extend/retract time: 2 ms. The Young's modulus of SLBs was determined by fitting the force *vs* tip-sample separation curves with the well-known Hertz model described in ref [7]. The fitting procedure was carried out with the JPK Data Processing software (version spm – 5.0.133). The AFM tip was approximated to a sphere with a curvature radius of 20 nm (Poisson ratio: 0.5). When operating in Force Volume mode, force maps consisting of 32×32 force distance curves were acquired point-by-point on scan areas of 5.0×5.0 μm^2. The maximum force load was 10 nN. Breakthrough forces were evaluated from the force-distance curves data sets using a home-built software.

Statistical data analysis. The bilayer Young's moduli shown in Figure 1A (main text) are reported as mean value ± standard error. For each lipid composition, results were averaged on at least 30000 curves deriving from at least 3 QI images. The bilayer breakthrough forces shown in Figure S5 are reported as mean values obtained from at least 3 Force Volume images. Errors were evaluated according to Student's statistics, assuming a confidence level of 95%.

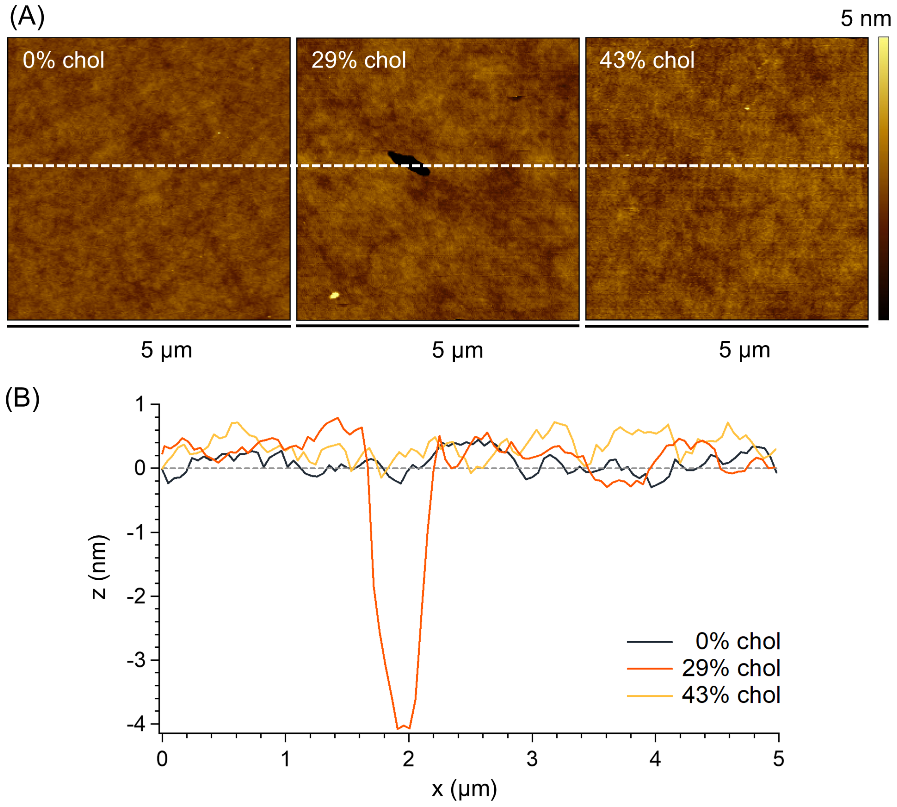

Figure S4. Tapping mode AFM topographic images of fluid DOPC SLBs with different cholesterol content, deposited on silicon. Images were acquired in PBS at room temperature. In general, for all samples in the 0 – 43 mol % chol range, the SLB surface was uniformly flat on a scale of tens of microns. (A) Representative images in the absence of cholesterol and in the case of medium or high cholesterol content (29 % and 43 %, respectively). (B) Height profiles of the SLBs taken along the white dashed lines in the images shown in panel (A); the minimum in the curve at 29 mol % chol corresponds to the deep bilayer hole (~4 nm) in the corresponding image.

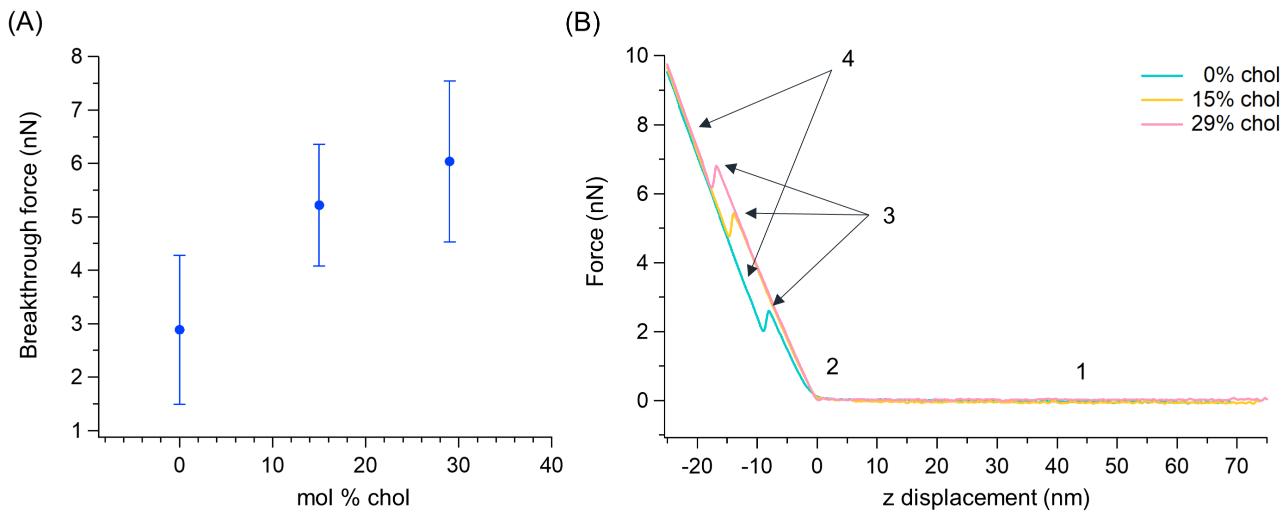

Figure S5. (A) The breakthrough force of DOPC SLBs increases with increasing membrane cholesterol content. (B) Typical force-distance curves recorded on SLBs when the AFM tip approaches the sample surface. The force is 0 nN when the tip is far from the SLB surface (1). After tip-bilayer contact, the force starts to increase (2). The breakthrough force is the force value at which the tip penetrates the bilayer (3). Immediately after the breakthrough event, the force dramatically decreases as a consequence of bilayer rupture. Subsequently, the force increases further due to the interaction between the tip and the solid substrate (4).

Fluorescence anisotropy assays

Steady-state fluorescence anisotropy was used to investigate the cholesterol-induced changes in membrane fluidity of DOPC vesicles. A Fluorolog-3 spectrofluorometer (Horiba Jobin-Ivon) equipped with polarizers and temperature control of the cell holder was employed. Measurements were performed at 20 °C under continuous magnetic stirring, in a quartz cuvette (Hellma, optical path length 10 mm × 4 mm) with sample volume of 850 µL. For each experiment, freshly extruded vesicle suspensions were diluted in PBS to a lipid concentration of 500 µM[8]. Before all acquisitions, polarizers were appropriately set on the excitation and the emission beams. The fluorescence intensity emitted by DPH fluorophores was detected after passing through the emission polarizer held vertical ($I_{\parallel}$) or horizontal ($I_{\perp}$) to the plane of polarization of the excitation beam. The fluorescence anisotropy (r) was then calculated as follows[9]:

$$r = \frac{I_{\parallel} - I_{\perp}}{I_{\parallel} + 2I_{\perp}} = \frac{I_{\parallel} - I_{\perp}}{I_{tot}} \qquad (1)$$

According to Equation 1, the fluorescence anisotropy of bilayer-embedded DPH molecules increases when their translational and orientational freedom decreases due to a reduction in the degree of membrane fluidity. All experiments were performed using excitation and emission slits with a bandpass of 4.5 nm; λ_{ex} and λ_{em} were set as 358 and 428 nm, respectively.

Statistical data analysis. Four replicates were performed for each experiment at varying membrane cholesterol. Uncertainties on mean values shown in Figure 2B (main text) were calculated using Student's statistics assuming a 95 % confidence level.

Computational methods

Computational model. In this study we used a coarse-grained (CG) model to represent the DOPC lipids, the cholesterol molecules and the functionalized amphiphilic AuNPs. Lipid membranes were modelled within the framework of the popular Martini CG force-field (2.2 version),[10] specifically suited for molecular dynamics simulations of biomolecular systems. We did not use the polarizable version of Martini water. A compatible model for the 70:30 MUS:OT NP with a gold core diameter of 2 nm has been previously developed[11] and used in several studies to study the NP-membrane interactions.[11–13] In this model, the Au core is made of 144 Au atoms and 60 S atoms (in an atomistic representation), while the MUS and OT ligands are represented by CG Martini beads, as described in previous works.[11,12]

MD simulation parameters. All the MD simulations were performed in the NPT ensemble: the velocity rescale thermostat[14] was used to keep constant temperature (T = 310 K, τ_T = 2 ps) while the Parrinello–Rahman barostat[15] (τ_P = 12 ps) was used to keep constant pressure (P = 1 bar) with semi-isotropic coupling. The time step used was 20 fs for all simulations. Both van der Waals and electrostatic interactions were treated with the cut-off method (r_c = 1.1 nm).[16]

Unbiased simulations settings. We constructed DOPC lipid bilayers containing different molar percentages of cholesterol (0%, 15%, 30%, 45%) with the Insane tool.[17] Each system has been solvated with Martini water beads and a physiological concentration (150 mM) of Na+ and Cl- ions. The size of the simulation box has been kept approximately constant in all the systems (~ 26x26x17 nm) to avoid spurious periodic boundary conditions effects on the membrane rigidity. After the insertion of a single NP in the water phase of each system (and Na+ counterions to keep it neutral), we let the system evolve in our unbiased MD simulations until the NP reached the adsorbed contact state. Then, starting from there, we performed 12 independent MD simulations for each system (changing the random seed to initialize the velocities) to measure the time interval spent by the NP in the hydrophobic state until the anchoring of the first ligand (Δt_{anchor}). These simulations have been limited to a maximum time length of 18 μs. In two simulations at 45% of cholesterol we could not reach the anchored state. In those cases, we used the time occurring between the hydrophobic contact state and the end of the simulations to estimate Δt_{anchor}. This choice led to a slight underestimation of the average Δt_{anchor} for the system with 45% of cholesterol, which reinforces even further the main

finding of the paper: increasing the percentage of cholesterol produces a slowdown of the NP incorporation into the membrane.

Well-Tempered Metadynamics parameters. We used the Well-Tempered Metadynamics (WT-MetaD) technique[18] to activate the process leading to the anchoring of the first MUS ligand on the opposite membrane leaflet and to measure the associated free energy barrier, following the same protocol adopted in previous works.[11,12] As collective variable, we used the distance along the z axis (perpendicular to the membrane plane) between the center of mass of the membrane and the charged terminal group of a MUS ligand. We used a Gaussian HEIGHT of 1.5 kJ mol^{-1}, a SIGMA of 0.1 nm and a BIASFACTOR of 20. The starting configurations of our WT-MetaD runs, in which the NP is in the hydrophobic contact state, were extracted from the unbiased MD simulations. To quantify the free energy barrier associated with the process, we followed the procedure described by Laio and Gervasio,[19] measuring the bias deposited until the time at which the transition had been completed.

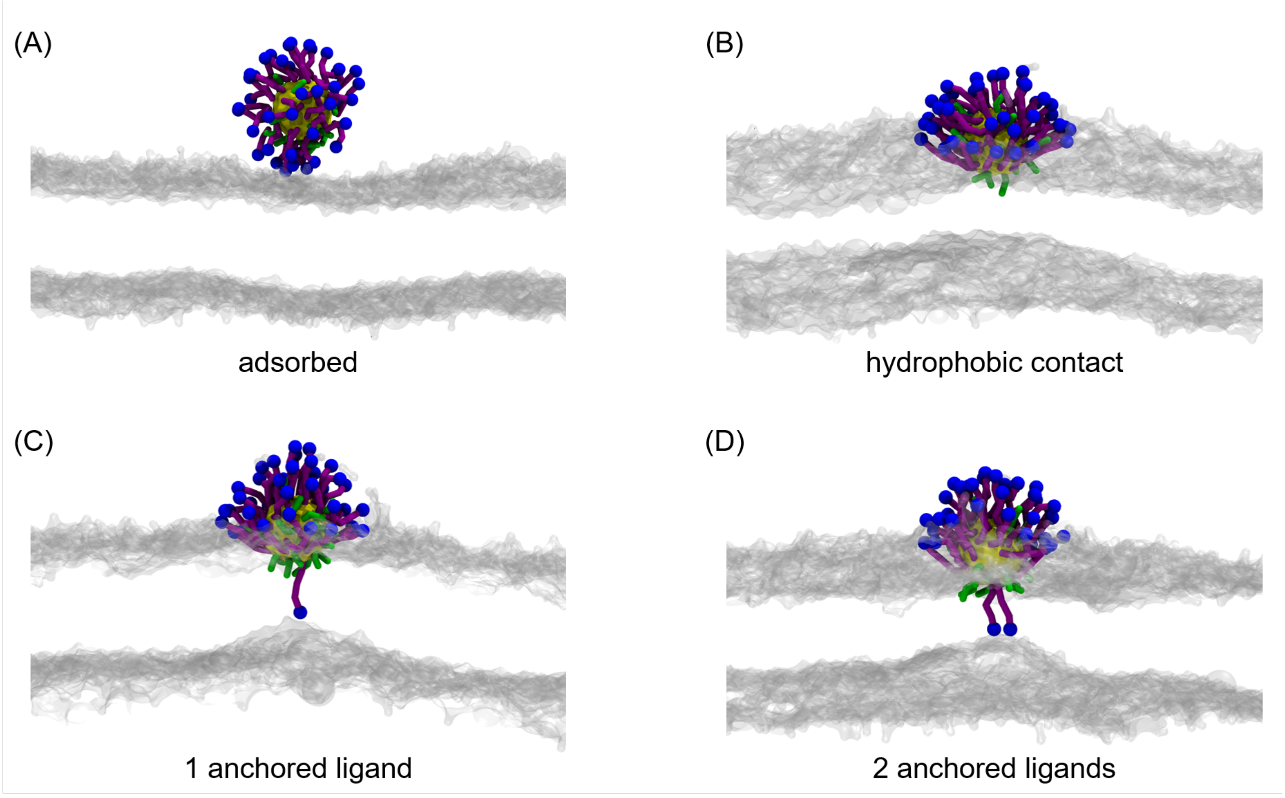

Figure S6. The multi-step process leading to the incorporation of an amphiphilic AuNPs into a lipid bilayer, as exemplified by CG simulation snapshots. A) The first step: the NP is adsorbed onto the lipid membrane (lipid heads shown in grey, lipid tails and water beads not shown for clarity). (B) The hydrophobic contact state: the NP starts penetrating into the hydrophobic core of the membrane, with the hydrophobic OT ligands (in green) coming in contact with the lipid tails. (C) The anchored state: the first MUS ligand (purple, with charged terminal in blue) has crossed the hydrophobic membrane core and is now anchored to the opposite leaflet heads. (D) With time, more and more ligands anchors on the opposite leaflet, and the incorporation of the NP becomes more and more stable.

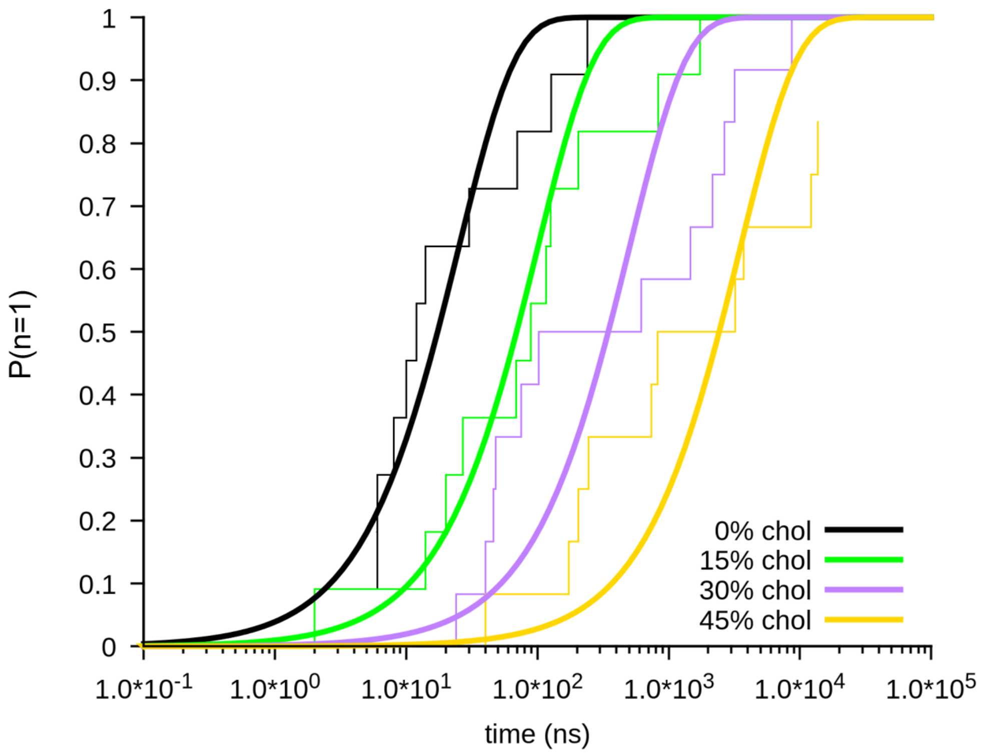

Figure S7. The cumulative distributions of the anchoring times (Δt_{anchor}) obtained from all the unbiased MD simulations at the different cholesterol molar percentages, represented as step functions (thin lines). Each step represent a Δt_{anchor} measured in a single simulation. The distributions are reasonably well fitted by cumulative Poissonian distributions (thick lines), with a characteristic transition time τ that increases exponentially with the cholesterol molar percentage.

Rescaling of the CG simulation time. Using the standard Martini version of the CG model (ΔG_{CG}) we observe spontaneous ligand anchoring in the pure DOPC membrane, suggesting a free energy barrier in the [0-12 kJ/mol] range (12 kJ/mol corresponds to 15% chol content). Let us assume a CG barrier of 6 kJ/mol. Atomistic force fields predict a barrier (ΔG_{atom}) at zero cholesterol content of at least ~47 kJ/mol.20 Considering this difference, it becomes clear that the characteristic time scale of the process is accelerated by several orders of magnitudes in the CG model. For the sake of completeness, we can estimate this acceleration factor with a straightforward, though approximated, calculation: we can assume that atomistic models provide the same transition rate for the ligand anchoring, k, as the experiments. We have:

$$\frac{k_{CG}}{k_{real}} = \frac{k_{CG}}{k_{atom}} \cong \exp\left(\frac{-(\Delta G_{CG} - \Delta G_{atom})}{k_B T}\right) \cong \exp(18.8) \cong 1.4\ 10^8$$

(where we also assumed equal prefactors). Observing the ligand anchoring in the microsecond time scale with our CG model means that the actual time scale of the process is in the time scale of 102 seconds.

QCM-D experiments

Dissipative QCM investigation was carried out in PBS using a QCM-Z500 microbalance (KSV Finland LLC) equipped with a thermostated flow chamber. To form homogeneous supported vesicle layers (SVLs), vesicles were deposited onto the surface of a gold-coated QCM sensors. These sensors contain a AT-cut disk-typed quartz crystal with standard resonance frequency of 5 MHz, a sensitivity coefficient (C_f) of 17.7 ng/(cm^2·Hz), and a piezoelectrically active surface of 78.5 mm^2. Before use, the sensors were subjected to UV/Ozone for at least 10 min. The monitoring of higher (3rd-11th) overtones was carried out for all experiments. In general, SVLs were used instead of more common SLBs[21,22] because vesicles containing increasing cholesterol content were unlikely to rupture on the sensor surface resulting in incomplete vesicle fusion.[23] The use of SLBs would therefore have led to poor reproducibility and comparability between results at different cholesterol percentages. Furthermore, we already showed in our previous work[13] that spontaneous absorption of amphiphilic MUS:OT NPs is facilitated in the free bilayer of lipid vesicles compared to that of SLBs.

Sample preparation. Figure S8 schematizes the steps of a typical experiment involving SVLs and NPs. For SVL formation, freshly extruded vesicle suspensions (Figure S3) were diluted in PBS (0.25 mg/mL) and then inserted into the QCM pre-chamber. After 10 min of equilibration at 22 °C, vesicle suspensions were injected into the QCM chamber. As shown in Figure 3A of the main text, SVL deposition occurred with different kinetics depending on the membrane cholesterol content. Before NP addition, each SVL was gently rinsed with fresh PBS (thermostated at 22 °C) to remove the excess of undeposited vesicles as well as any vesicle loosely attached to the SVL surface (Figure 4A, main text). In general, SVLs were highly stable after deposition and rinse. For NP addition, a fixed NP volume was taken from a stock solution in water (0.6 mg/mL) and diluted in PBS (2 mL) to achieve a lipid/NP molar ratio of ~1600 with respect to the vesicle concentration previously injected to form the SVL (0.25 mg/mL). This ratio is nominal since excess vesicles were rinsed away before NP addition (Figure S8, step 2); the real lipid/NP value during SVL-NP incubation is therefore lower. Each NP dispersion in PBS was then sonicated for a few minutes in an ultrasonic bath and finally inserted into the pre-chamber. After 10 min of equilibration at 22 °C, NPs were injected into the chamber and let to interact with the preformed SVL for at least 20 h at 22 °C (Figure 4A, main text). After a few hours with a sampling interval of 1 s, a point average every 10 s was selected. The following day, the NP-SVL complex was gently rinsed with PBS before the end of the recording. In all cases, no mass losses were recorded after buffer exchange (Figure 4A, main text).

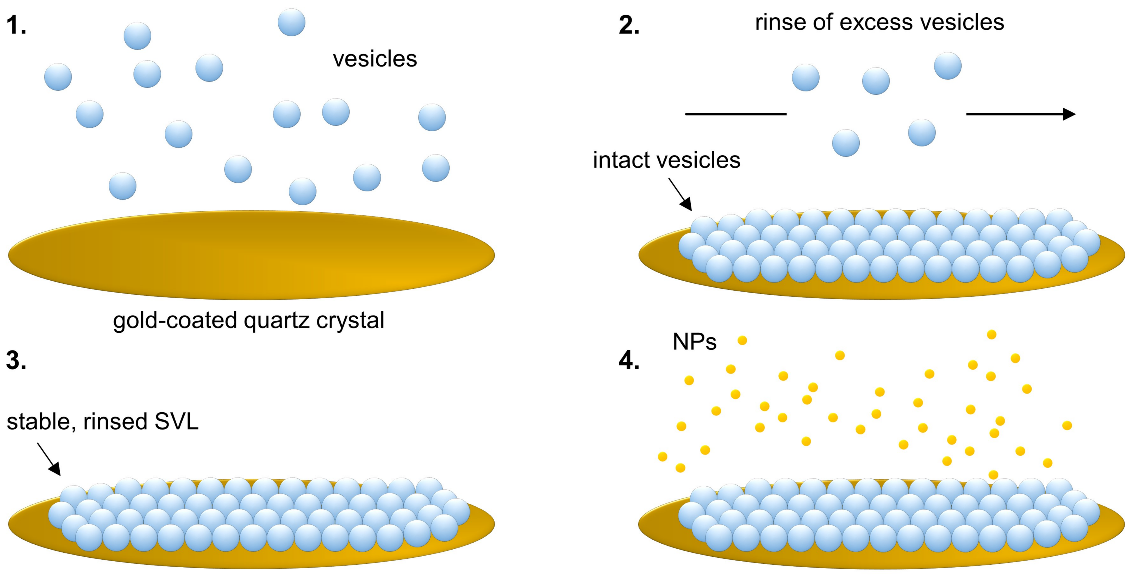

Figure S8. Scheme (not to scale) illustrating the timeline of the QCM-D experiments shown in this study. Initially, freshly extruded lipid vesicles (0.25 mg/ml) are injected into the QCM chamber previously filled with PBS (**1**). Over the course of several hours, the vesicles are deposited on the surface of the gold-coated quartz crystal sensor, where they accumulate intact and form a vesicular layer. After rinsing with fresh PBS to remove undeposited vesicles, (**2**), the SVL is allowed to interact with NPs for at least 20 hours (**3** – **4**). Before the end of the QCM-D recording, the NP-SVL complex is rinsed with fresh PBS to wash the NPs not penetrated into the vesicle bilayer. Experiments are performed at 22°C in PBS.

Details on NP molecular weight calculation. To calculate the lipid/NP ratio used for QCM experiments, the mean NP molecular weight (MW_{NP} 68689 g/mol) was estimated considering an Au density of 34.377 atoms/nm^3 and a ligand density of 4.775 molecules/nm^2. These values derive from the NP model reported by Lopez-Acevedo et al.[24].

Data processing. QCM-D results after SVL stabilization (and rinsing) were processed using the viscoelastic Voigt model described in ref[25]. This modeling provides information on membrane viscoelastic properties, including layer density, thickness, viscosity, and elasticity. In particular, data processing was carried out by fitting experimental Δf and ΔD data (from at least 4 harmonics) to the Voigt model included in the KSV QCM Impedance Analyzer Software (version 2.0)[26] and using the SVL thickness (L, nm), density (ρ, g/cm^3), viscosity (η, Ns/m^2), and shear modulus of elasticity (G', MPa) as fitting parameters. In recursive fitting procedures, the SVL viscoelastic properties were obtained starting from viscosity and elastic modulus values derived from literature data[27]. Density and thickness results are reported in Figure 3B of the main text, whereas final viscosity and elastic modulus values are included in Figure S10.

After NP-SVL interaction, QCM-D results were processed in the assumption of a modified Sauerbrey model to include the change in viscosity typical of a viscoelastic layer (Figure S10):

$$\Delta m = -C_f \frac{\Delta \mathrm{f}}{n^{\frac{1}{2}}} \qquad (2)$$

where: n is the overtone number at which the crystal is driven (in the field of acoustic waves, only odd harmonics are measured), $\frac{\Delta \mathrm{f}}{n^{\frac{1}{2}}}$ (Δf_{norm}) is the observed frequency shift (Hz) normalized by $n^{\frac{1}{2}}$ instead of the canonical n, C_f is the previously reported sensitivity coefficient of the quartz crystal sensor, and Δm is the change in mass per unit area of the piezoelectrically active surface (ng/cm^2). By applying Equation 2 to Δf values after SVL rinsing and after maximum NP uptake, it was possible to calculate the mass changes due to the SVL formation (m_{SVL}) and NPs (m_{NP}), respectively (Figure 4B, main text). To validate such data processing, the Voigt model was applied to determine the SVL thickness (L, nm) and density (ρ, g/cm^3) before and after interaction with NPs. The mass per unit area adsorbed onto the sensor surface after SVL formation (m_{SVL}) and after maximum NP uptake (m_{NP}) (Figure 4B, main text) was then derived from thickness and density data ($m = L\,\rho$).

Statistical data analysis. At least two replicates were performed for each QCM experiment. Uncertainties on the mass changes shown in Figure 4B of the main text were processed by averaging the results obtained from the overtones of different replicates and by applying the Student's statistics (95 % confidence level, N≥10). The same statistics was used to calculate the vertical error bars referring to the reduction (%) in NP uptake shown in the same figure (N=18). Only for the SVL viscoelastic properties shown in Figure 3B (main text) and Figure S10, error bars correspond to standard deviation.

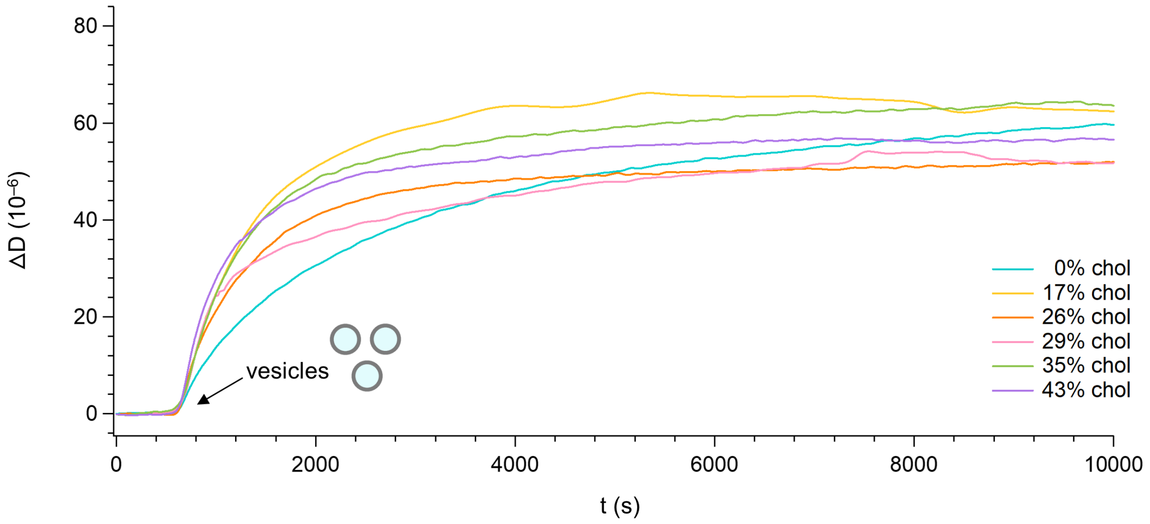

Figure S9. Asymptotic dissipation shifts (5th overtone) recorded after the injection in the sensor chamber of lipid vesicles with varying cholesterol molar percentage. The increase in dissipation is typical of the deposition of viscoelastic layers on the QCM sensor surface. An average over 20 points was applied.

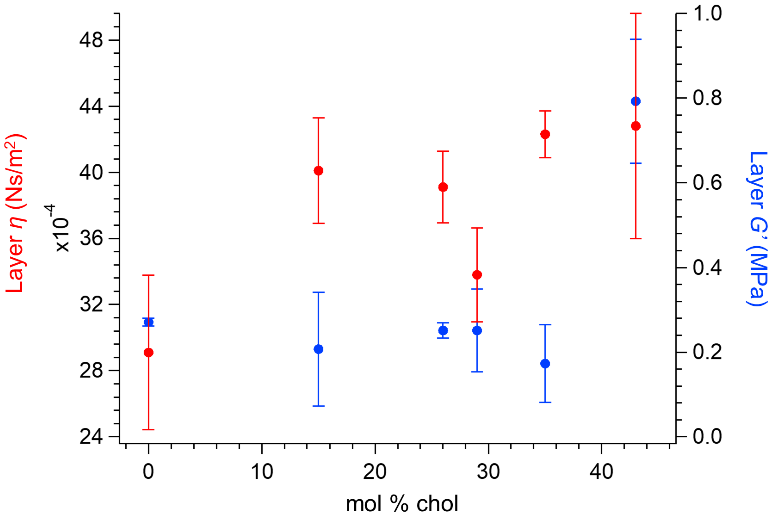

Figure S10. Layer viscosity (η) and shear modulus of elasticity (G') derived from the Voigt modeling included in the KSV QCM Impedance Analyzer Software.

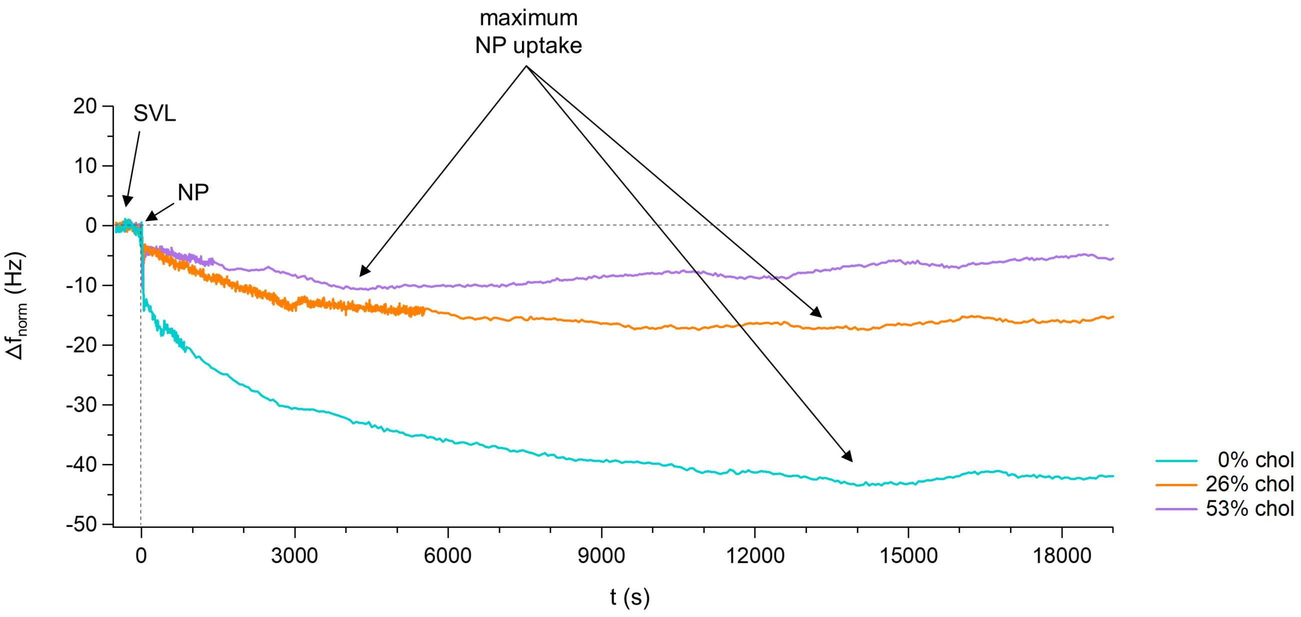

Figure S11. Representative curves showing the frequency shifts (3rd overtone) recorded after NP addition (t = 0 s) on preformed SVLs at different molar percentages of membrane cholesterol. In the initial part of the curve, which is thicker, the sampling interval corresponded to 1 s, while in the following part it was decreased to 10 s. The entire QCM experiment recorded at 17 mol % cholesterol is shown in Figure S12.

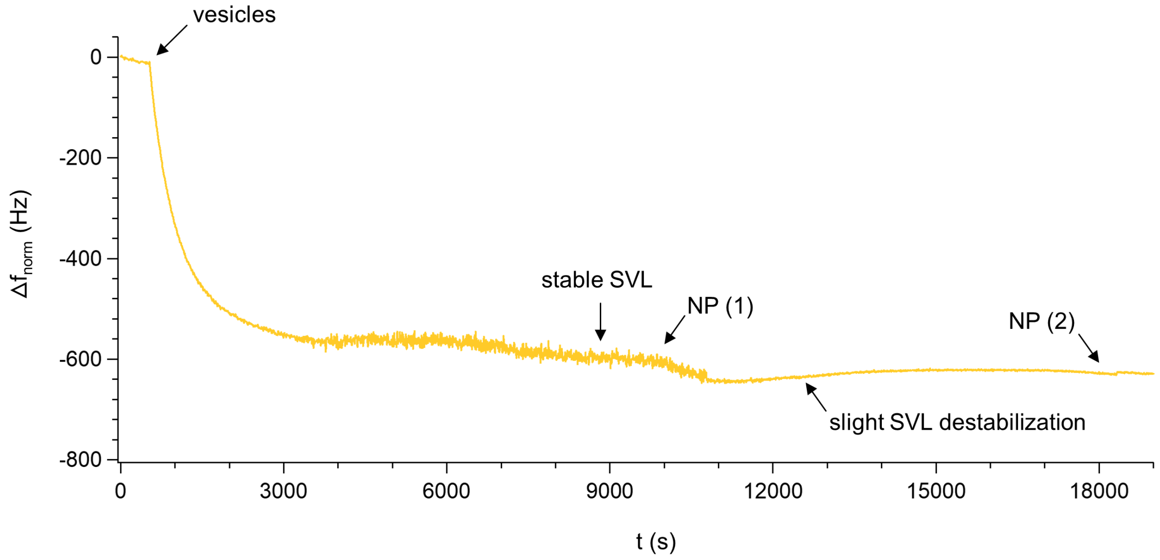

Figure S12. Frequency shift (3rd overtone) recorded during the QCM-D experiment at 17 mol % cholesterol (in the thickest parts of the curve the sampling interval corresponds to 1 s, while in the others to 10 s). A second NP addition – identical to the first one – was performed to test whether saturation of the SVL portion exposed to NPs had been reached. Whereas a clear decrease in resonance frequency was observed after the first NP injection, the signal remained unperturbed after the second. This control measurement suggested that the amount of NPs inserted in QCM experiments (see above) was sufficient to reach membrane saturation.

Quantification of the lipid/NP ratio. The calculation of lipid/NP molar ratios was made in the same assumptions considered for the viscoelastic model, i.e. assuming no water loss from the vesicles

during NP adsorption and invariance of vesicle number (see main text). The density ρ (g/cm^3) and thickness L (nm) of the viscoelastic vesicle layers (see Figure 3B, main text) were used to calculate the total mass adsorbed onto the sensor before the SVL–NP interaction, assuming complete coverage of the sensor surface (0.78 cm^2). The mass change induced by NP uptake and extracted from the viscoelastic model was used to determine the mass – and thus the number – of the embedded NPs (MW_{NP} 68689 g/mol, see above). The number of adsorbed vesicles was estimated by dividing the total area of the sensor into square boxes of size L to account for the volume of free water between vesicles. Finally, the number of lipid molecules per vesicle was calculated using the area-per-molecule values reported in the literature for the same membrane compositions[24] and considering that the surface of the vesicle prone to interact with NPs is only the hemisphere facing the buffer. The calculation of the average lipid/NP ratios thus obtained is shown in Table S3. The starting value for pure DOPC (73 lipid/NP) is in complete agreement with our previous investigation quantifying the uptake of the same amphiphilic NPs into another fluid lipid membrane (79 lipids/NP)[13].

Table S3. Estimated average lipid/NP ratios derived from QCM-D measurements.

mol % chol	*lipid/NP ratios*
0	**73**
17	**113**
30	**266**
33	**205**
40	**839**
50	**560**

References

(1) Guven, Z. P.; Jacob Silva, P. H.; Luo, Z.; Cendrowska, U. B.; Gasbarri, M.; Jones, S. T.; Stellacci, F. Synthesis and Characterization of Amphiphilic Gold Nanoparticles. *Journal of Visualized Experiments* **2019**, *2019* (149), 1–11. https://doi.org/10.3791/58872.

(2) Yang, Y.; Serrano, L. A.; Guldin, S. A Versatile AuNP Synthetic Platform for Decoupled Control of Size and Surface Composition. *Langmuir* **2018**, *34* (23), 6820–6826. https://doi.org/10.1021/acs.langmuir.8b00353.

(3) Eriksson, E. K.; Agmo Hernández, V.; Edwards, K. Effect of Ubiquinone-10 on the Stability of Biomimetic Membranes of Relevance for the Inner Mitochondrial Membrane. *Biochimica et Biophysica Acta - Biomembranes* **2018**, *1860* (5), 1205–1215. https://doi.org/10.1016/j.bbamem.2018.02.015.

(4) ThermoFisher Scientific. Probes for Lipids and Membranes. *Molecular Probes Handbook* **2010**, *76* (11), 547–589. https://doi.org/10.1134/S0006297911110101.

(5) Ibarguren, M.; Alonso, A.; Tenchov, B. G.; Goñi, F. M. Quantitation of Cholesterol Incorporation into Extruded Lipid Bilayers. *Biochimica et Biophysica Acta - Biomembranes* **2010**, *1798* (9), 1735–1738. https://doi.org/10.1016/j.bbamem.2010.06.004.

(6) Hutter, J. L.; Bechhoefer, J. Calibration of Atomic-force Microscope Tips. *Review of Scientific Instruments* **1993**, *64* (7), 1868–1873. https://doi.org/10.1063/1.1143970.

(7) Neumann, T. Determining the Elastic Modulus of Biological Samples Using Atomic Force Microscopy. *JPK Instrument - Application Note* 1–9.

(8) Alves, A. C.; Ribeiro, D.; Horta, M.; Lima, J. L. F. C.; Nunes, C.; Reis, S. The Daunorubicin Interplay with Mimetic Model Membranes of Cancer Cells: A Biophysical Interpretation. *Biochimica et Biophysica Acta - Biomembranes* **2017**, *1859* (5), 941–948. https://doi.org/10.1016/j.bbamem.2017.01.034.

(9) Lakowicz, J. R. *Principles of Fluorescence Spectroscopy*, 3rd ed.; Springer New York, 2006. https://doi.org/10.1007/978-0-387-46312-4.

(10) Marrink, S. J.; Risselada, H. J.; Yefimov, S.; Tieleman, D. P.; de Vries, A. H. The MARTINI Force Field: Coarse Grained Model for Biomolecular Simulations. *The Journal of Physical Chemistry B* **2007**, *111* (27), 7812–7824. https://doi.org/10.1021/jp071097f.

(11) Simonelli, F.; Bochicchio, D.; Ferrando, R.; Rossi, G. Monolayer-Protected Anionic Au Nanoparticles Walk into Lipid Membranes Step by Step. *Journal of Physical Chemistry Letters* **2015**, *6* (16), 3175–3179. https://doi.org/10.1021/acs.jpclett.5b01469.

(12) Salassi, S.; Simonelli, F.; Bochicchio, D.; Ferrando, R.; Rossi, G. Au Nanoparticles in Lipid Bilayers: A Comparison between Atomistic and Coarse-Grained Models. *Journal of Physical Chemistry C* **2017**, *121* (20), 10927–10935. https://doi.org/10.1021/acs.jpcc.6b12148.

(13) Canepa, E.; Salassi, S.; De Marco, A. L.; Lambruschini, C.; Odino, D.; Bochicchio, D.; Canepa, F.; Canale, C.; Dante, S.; Brescia, R.; Stellacci, F.; Rossi, G.; Relini, A. Amphiphilic Gold Nanoparticles Perturb Phase Separation in Multidomain Lipid Membranes. *Nanoscale* **2020**, *12* (38), 19746–19759. https://doi.org/10.1039/d0nr05366j.

(14) Bussi, G.; Donadio, D.; Parrinello, M. Canonical Sampling through Velocity Rescaling. *Journal of Chemical Physics* **2007**, *126* (1), 014101. https://doi.org/10.1063/1.2408420.

(15) Parrinello, M.; Rahman, A. Polymorphic Transitions in Single Crystals: A New Molecular Dynamics Method. *Journal of Applied Physics* **1981**, *52* (12), 7182–7190. https://doi.org/10.1063/1.328693.

(16) De Jong, D. H.; Baoukina, S.; Ingólfsson, H. I.; Marrink, S. J. Martini Straight: Boosting Performance Using a Shorter Cutoff and GPUs. *Computer Physics Communications* **2016**, *199*, 1–7. https://doi.org/10.1016/j.cpc.2015.09.014.

(17) Wassenaar, T. A.; Ingólfsson, H. I.; Böckmann, R. A.; Tieleman, D. P.; Marrink, S. J. Computational Lipidomics with Insane: A Versatile Tool for Generating Custom Membranes for Molecular Simulations. *Journal of Chemical Theory and Computation* **2015**, *11* (5), 2144–2155. https://doi.org/10.1021/acs.jctc.5b00209.

(18) Barducci, A.; Bussi, G.; Parrinello, M. Well-Tempered Metadynamics: A Smoothly Converging and Tunable Free-Energy Method. *Physical Review Letters* **2008**, *100* (2), 020603. https://doi.org/10.1103/PhysRevLett.100.020603.

(19) Laio, A.; Gervasio, F. L. Metadynamics: A Method to Simulate Rare Events and Reconstruct the Free Energy in Biophysics, Chemistry and Material Science. *Reports on Progress in Physics* **2008**, *71* (12), 126601. https://doi.org/10.1088/0034-4885/71/12/126601.

(20) Van Lehn, R. C.; Alexander-Katz, A. Grafting Charged Species to Membrane-Embedded Scaffolds Dramatically Increases the Rate of Bilayer Flipping. *ACS Central Science* **2017**, acscentsci.6b00365. https://doi.org/10.1021/acscentsci.6b00365.

(21) Lochbaum, C. A.; Chew, A. K.; Zhang, X.; Rotello, V.; Van Lehn, R. C.; Pedersen, J. A. Lipophilicity of Cationic Ligands Promotes Irreversible Adsorption of Nanoparticles to Lipid Bilayers. *ACS Nano* **2021**, *15* (4), 6562–6572. https://doi.org/10.1021/acsnano.0c09732.

(22) Troiano, J. M.; Olenick, L. L.; Kuech, T. R.; Melby, E. S.; Hu, D.; Lohse, S. E.; Mensch, A.

C.; Dogangun, M.; Vartanian, A. M.; Torelli, M. D.; Ehimiaghe, E.; Walter, S. R.; Fu, L.; Anderton, C. R.; Zhu, Z.; Wang, H.; Orr, G.; Murphy, C. J.; Hamers, R. J.; Pedersen, J. A.; Geiger, F. M. Direct Probes of 4 Nm Diameter Gold Nanoparticles Interacting with Supported Lipid Bilayers. *Journal of Physical Chemistry C* **2015**, *119* (1), 534–546. https://doi.org/10.1021/jp512107z.

(23) Tabaei, S. R.; Jackman, J. A.; Kim, S. O.; Liedberg, B.; Knoll, W.; Parikh, A. N.; Cho, N. J. Formation of Cholesterol-Rich Supported Membranes Using Solvent-Assisted Lipid Self-Assembly. *Langmuir* **2014**, *30* (44), 13345–13352. https://doi.org/10.1021/la5034433.

(24) Lopez-Acevedo, O.; Akola, J.; Whetten, R. L.; Grönbeck, H.; Häkkinen, H. Structure and Bonding in the Ubiquitous Icosahedral Metallic Gold Cluster Au 144 (SR) 60. *The Journal of Physical Chemistry C* **2009**, *113* (13), 5035–5038. https://doi.org/10.1021/jp8115098.

(25) Voinova, M. V; Rodahl, M.; Jonson, M.; Kasemo, B. Viscoelastic Acoustic Response of Layered Polymer Films at Fluid-Solid Interfaces: Continuum Mechanics Approach. *Physica Scripta* **1999**, *59* (5), 391–396. https://doi.org/10.1238/physica.regular.059a00391.

(26) Viitala, T.; Hautala, J. T.; Vuorinen, J.; Wiedmer, S. K. Structure of Anionic Phospholipid Coatings on Silica by Dissipative Quartz Crystal Microbalance. *Langmuir* **2007**, *23* (2), 609–618. https://doi.org/10.1021/la060923t.

(27) Reviakine, I.; Rossetti, F. F.; Morozov, A. N.; Textor, M. Investigating the Properties of Supported Vesicular Layers on Titanium Dioxide by Quartz Crystal Microbalance with Dissipation Measurements. *The Journal of Chemical Physics* **2005**, *122* (20), 204711. https://doi.org/10.1063/1.1908500.
